# Accelerating protein release from microparticles for regenerative medicine applications

**DOI:** 10.1016/j.msec.2013.02.020

**Published:** 2013-07-01

**Authors:** Lisa J. White, Giles T.S. Kirby, Helen C. Cox, Roozbeh Qodratnama, Omar Qutachi, Felicity R.A.J. Rose, Kevin M. Shakesheff

**Affiliations:** School of Pharmacy, University of Nottingham, Nottingham NG7 2RD, UK

**Keywords:** Poly (d,l-lactic -*co*-glycolic acid) (PLGA), Microparticles, Microspheres, Controlled release, Double emulsion solvent evaporation, Growth factor delivery

## Abstract

There is a need to control the spatio-temporal release kinetics of growth factors in order to mitigate current usage of high doses. A novel delivery system, capable of providing both structural support and controlled release kinetics, has been developed from PLGA microparticles. The inclusion of a hydrophilic PLGA–PEG–PLGA triblock copolymer altered release kinetics such that they were decoupled from polymer degradation. A quasi zero order release profile over four weeks was produced using 10% w/w PLGA–PEG–PLGA with 50:50 PLGA whereas complete and sustained release was achieved over ten days using 30% w/w PLGA–PEG–PLGA with 85:15 PLGA and over four days using 30% w/w PLGA–PEG–PLGA with 50:50 PLGA. These three formulations are promising candidates for delivery of growth factors such as BMP-2, PDGF and VEGF. Release profiles were also modified by mixing microparticles of two different formulations providing another route, not previously reported, for controlling release kinetics. This system provides customisable, localised and controlled delivery with adjustable release profiles, which will improve the efficacy and safety of recombinant growth factor delivery.

## Graphical abstract

**Figure:**
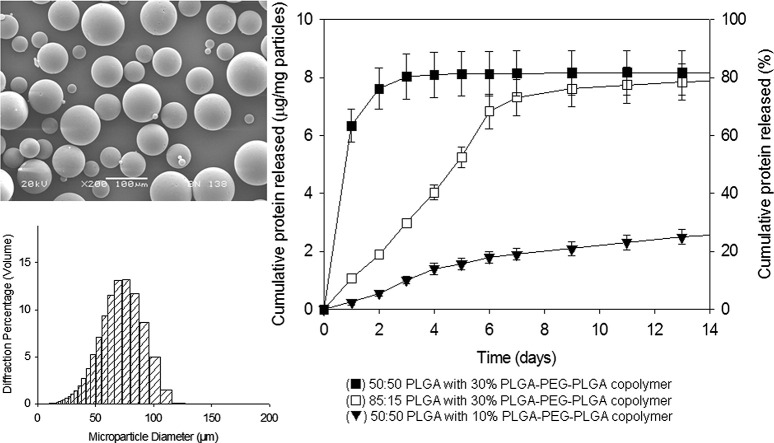

**Highlights:**

► A new delivery system providing controlled release kinetics has been developed. ► Inclusion of hydrophilic PLGA–PEG–PLGA decoupled release kinetics from degradation. ► Using 10% triblock copolymer produced quasi zero order release over four weeks. ► Mixing microparticle formulations provided another route for controlling release. ► This system provides customisable, localised and controlled delivery of growth factors.

## 1 Introduction

In bone and cartilage repair, a complex cascade of biological events is controlled by growth factor signalling at injury sites, promoting progenitors and inflammatory cells to migrate and trigger healing processes [1]. Delivery of growth factors such as bone morphogenetic proteins (BMPs), vascular endothelial growth factor (VEGF) and transforming growth factor beta (TGF-β) can stimulate cellular adhesion, proliferation and differentiation and form an attractive therapeutic strategy to promote endogenous repair [2]. Bone morphogenetic proteins, in particular, have been extensively studied [3,4] and two recombinant human bone morphogenetic proteins (rhBMPs), rhBMP-2 (Infuse™, Medtronic Sofamor Danek Inc.) and rhBMP-7 (OP-1™, Stryker Biotech) have been approved by regulatory bodies for the treatment of non-union bone defects, open tibial fractures and spinal fusion [5–9]. However, administration of rhBMPs in orthopaedic applications is complicated by their short biological half lives, localised action and rapid clearance [10,11]. This has resulted in the clinical use of supraphysiologic concentrations of rhBMPs; currently a dose of ~ 60 μM BMP-2 is delivered (via a purified type I collagen matrix) which greatly exceeds the effective physiological range of 1–30 nM [12].

In 2008 the US Food and Drug Administration (FDA) issued a Public Health Notification of life threatening complications associated with rhBMP-2 use; these included complications associated with swelling of neck and throat tissue in anterior cervical disectomy and fusion (ACDF) procedures, as described by Perri and associates [13]. Further to this, a recent critical review has outlined the risks associated with clinical BMP-2 usage in spinal surgery and highlighted problems encountered with high doses; an increased risk of malignancy was indicated with the use of AMPLIFY in posterolateral spinal fusion [14]. Sustained delivery of rhBMP-2 could therefore address the need to mitigate these high doses and corresponding health and cost implications [15]. These implications emphasise the need for strategies to improve the efficacy and safety of recombinant growth factor delivery.

Control of delivery can be achieved by incorporating the growth factor into a biomaterial carrier system; this strategy may also prevent growth factor degradation as the biomaterial vehicle can provide protection from proteolytic enzymes at the target site [16]. Incorporation strategies include non-covalent mechanisms, such as physical entrapment, surface adsorption and complexation, or covalent immobilisation on or into the delivery vehicle. Spatio-temporal control of growth factor delivery has been achieved via encapsulation within polymeric microparticles as this can provide independent regulation of duration and availability of soluble factors whilst limiting dose and undesirable side effects [17]. Additionally, scaffolds formed from microparticles can host tissue formation whilst maintaining desired local growth factor concentrations [18]. Homo and copolymers of lactide and glycolide have found particular application as controlled delivery vehicles as they are FDA approved for various clinical functions [19], degrade in vivo into natural products (lactic and glycolic acid) that are processed by normal metabolic pathways [20–22] and have tuneable physico-chemical properties [16].

Herein we describe the development of a novel growth factor delivery system with kinetics of release faster than scaffold degradation. This system has the capability to provide both structural support and the release of growth factors in a controlled spatio-temporal manner, when injected or inserted into a defect site. The delivery system is comprised of large (~ 100 μm) microparticles fabricated from poly (d,l-lactic -*co*-glycolic acid) (PLGA) and an ‘in-house’ produced triblock copolymer, containing poly(ethylene glycol) (PEG): PLGA–PEG–PLGA [23]. Addition of the triblock copolymer accelerates release kinetics such that the encapsulated growth factor can be released prior to microparticle degradation. This localised growth factor delivery system can also provide a supportive structure and we anticipate that the size of the microparticles will ensure that the void filled by the delivery system will confer mechanical support whilst the pore space between the microparticles contributes to enhanced cell infiltration and proliferation. We report on the characterisation of the size, morphology and release kinetics of this novel delivery system with initial in vitro release studies undertaken using lysozyme, a suitable model protein for rh-BMP2. This system provides customisable, localised and controlled delivery with adjustable release profiles, which we anticipate will improve the efficacy and safety of therapeutic growth factor delivery.

## 2 Materials and methods

### 2.1 Materials

Poly(vinyl alcohol) (PVA, molecular weight: 13,000–23,000 Da, 87–89% hydrolysed), lysozyme from chicken egg white, human serum albumin (HSA), polyethylene glycol (PEG, molecular weight: 1500 Da), stannous octoate/Tin(II) 2-ethylhexanoate (Sn(Oct)_2_), dimethyl sulphoxide (DMSO), sodium hydroxide (NaOH), sodium dodecyl sulphate (SDS) and *Micrococcus lysodeikticus* were obtained from Sigma–Aldrich, Dorset, UK. Poly (d,l-lactide-*co*-glycolide) (PLGA) polymers with lactide:glycolide ratios of 50:50 and 85:15 (PLGA 85:15 DLG 4A 56 kDa and PLGA 50:50 DLG 4.5A 59 kDa) were purchased from Surmodics, Birmingham, USA. d,l-Lactide and glycolide monomers were obtained from Lancaster Synthesis, Ward Hill, MA, USA and PURAC, Gorinchem, Netherlands, respectively. The Pierce Micro bicinchoninic acid (BCA) protein assay kit and HPLC grade solvents (dichloromethane (DCM) and acetone) were purchased from Fisher Scientific UK Ltd, Loughborough, UK. Recombinant human BMP-2 (BMP-2) was purchased from Professor Walter Sebald (University of Wurzburg, Germany).

### 2.2 Methods

#### 2.2.1 Triblock copolymer synthesis, purification and characterisation

Synthesis of the PLGA–PEG–PLGA triblock copolymer occurred by ring opening polymerisation of the d,l-lactide and glycolide monomers in the presence of PEG and the Sn(Oct)_2_ catalyst under a dry nitrogen atmosphere, as previously described [24,25]. Prior to polymerisation, PEG was first dried in the reaction vessel under vacuum and stirring at 120 °C for 3 h. The temperature was then raised to 150 °C, the monomers added to the vessel and the reaction mixture allowed to equilibrate under a dry nitrogen atmosphere. After 30 min, the Sn(Oct)_2_ was added and the reaction was allowed to proceed for 8 h. The resultant copolymer was dissolved and precipitated in water in order to remove un-reacted monomers. The triblock copolymer was then dried under vacuum to remove residual water and stored at − 20 °C until required.

Nuclear Magnetic Resonance (NMR) analysis of the copolymer was undertaken using a Bruker DPX-300 Spectrometer (300 MHz) with deuterated choloroform (CDCl_3_) as the solvent. A tetramethylsilane (TMS) signal was taken as the zero chemical shift. As described by Hou et al. [25], the composition of the copolymer was determined by ^1^H NMR by integrating the signals pertaining to each monomer, i.e. peaks from CH_2_ of ethylene glycol and glycolide and CH and CH_3_ from d,l-lactide. The molecular weight and polydispersity index of the copolymer was determined using Gel Permeation Chromatography (PL-GPC 120, Polymer Labs) with differential refractometer detection. Tetrahydrofuran (THF) was employed as an eluent, with two columns (30 cm, PolarGel-M) in series calibrated against polystyrene standards. Characteristics of the triblock copolymer are presented in Table 1.

#### 2.2.2 Microparticle preparation

Poly (d,l-lactide-*co*-glycolide) microparticles were formed using a water-in-oil-in-water (w/o/w) emulsion method as previously described [23]. Briefly, an aqueous solution of human serum albumin (HSA) and lysozyme (or BMP-2) was added to a solution of PLGA and PLGA–PEG–PLGA in dichloromethane. These phases were homogenised for two minutes at 4000 rpm in a Silverson L5M homogeniser (Silverson Machines, UK) to form the water-in-oil emulsion. This primary emulsion was transferred to 200 ml 0.3% (w/v) PVA solution and was homogenised for a second time at 2000 rpm for two minutes. The resultant double emulsion was stirred at 300 rpm on a Variomag 15-way magnetic stirrer for a minimum of 4 h to facilitate DCM evaporation. Microparticles were then filtered, washed and lyophilized (Edwards Modulyo, IMA Edwards, UK) until dry.

The triblock copolymer was added to PLGA to provide weight percentages of 0, 10, and 30% (w/w) of the 1 g total mass (in 5 ml DCM). Lysozyme (or BMP-2) and HSA were prepared at a ratio of 1:9 for a 1% w/w loading in the microparticles. That is, for 1 g of polymer, 1 mg of lysozyme (or BMP-2) and 9 mg of HSA were dissolved in 100 μl distilled water. Control particles were manufactured without protein, using 100 μl distilled water in the primary emulsion.

Lysozyme was selected as an appropriate model for BMP-2 in this work, due to the similarity of isoelectric points (lysozyme: 9, BMP-2: > 8.5) and molecular weights (lysozyme: 14 kD, BMP-2: 26 kD) [26].

#### 2.2.3 Microparticle characterisation — size, morphology and protein entrapment

Microparticles (50 mg/ml in double deionised water) were sized using a laser diffraction method (Coulter LS230, Beckman Coulter, UK) with agitation to prevent particle settling. To assess the morphology of the microparticles, thin layers of freeze dried microparticles were adhered to an adhesive stub and gold sputter coated for four minutes at 30 mA (Balzers SCD 030 gold sputter coater, Balzers, Liechenstein). Microparticles were imaged using a JSM 6060LV Scanning Electron Microscope (SEM) (JEOL, Welwyn Garden City, UK) with the accelerating voltage set to 10 kV.

Measurement of the encapsulation efficiency of protein within the microparticles was undertaken as previously reported [23]; the method was a modification of techniques proposed by Sah [27] and Morita et al. [28]. Briefly, 10 mg of PLGA microspheres were added to 750 μl DMSO and left at room temperature for one hour; 2150 μl of 0.02% (w/v) SDS in 0.2 M NaOH was then added for further one hour incubation. The Micro BCA protein assay kit was used to ascertain the total protein content and compared against a standard curve of HSA/lysozyme conducted at the same time. Sample (150 μl) and BCA working reagent (150 μl) were mixed and incubated for 2 h at 37 °C and the absorbance at 562 nm measured using a plate reader (Infinite M200, Tecan UK Ltd., Reading, UK).

#### 2.2.4 Protein release from microparticles

Aliquots (100 mg) of the microparticles (triplicate samples from each batch) were suspended in 3 ml phosphate buffered saline (PBS, pH 7.4); samples were gently rocked on a 3-dimensional shaker (Gyrotwister, Fisher Scientific UK Ltd) at 5 rpm in a humidified incubator at 37 °C. At defined time intervals, the PBS was removed from the microparticles and replaced with 3 ml fresh PBS; all liquid above the particles was collected without removing particles. The removed supernatants were stored frozen until required and were then assayed for total protein content using the Micro BCA assay kit with a standard curve of HSA/lysozyme (0–40 μg/ml).

The activity of lysozyme in collected supernatants was measured using the method described by Bezemer et al. [29] and Sohier et al. [30] with slight modifications. The change in turbidity, that occurred in an *M. lysodeikticus* solution when lysozyme lysed the 1,4 glycosidic bond within the cell wall of the bacteria, was measured. *M. lysodeikticus* suspension (100 μl of 2.3 mg/ml) was added to 150 μl of the release supernatant. The change in turbidity over a one minute period was determined by evaluation of the absorbance at 450 nm, measured using a plate reader (Infinite M200, Tecan UK Ltd., Reading, UK). Absorbance values were correlated to a standard curve of HSA/lysozyme (0–10 μg/ml) in order to determine the activity of lysozyme.

## 3 Results

### 3.1 Size, morphology and protein entrapment

Polymer microparticles with encapsulated HSA and lysozyme were fabricated using the double emulsion method described above. Six different formulations were manufactured by varying the lactide:glycolide ratio (50:50 and 85:15) and by incorporating three different percentage weights of the PLGA–PEG–PLGA triblock copolymer (0%, 10% and 30%). The size, morphology and in vitro release profile of microparticles from each formulation were characterised.

**Fig. 1:**
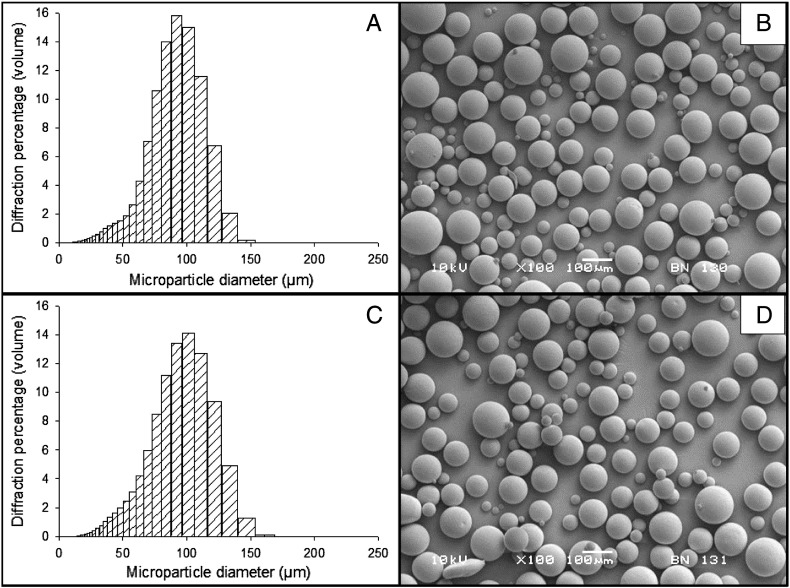
Morphology of microparticles formulated from 85:15 PLGA with 10% w/w PLGA–PEG–PLGA, with size distributions and SEM images of the blank (no protein) microparticles (A and B) and protein loaded (1% w/w) microparticles (C and D).

Representative images of microparticle morphology are shown in Fig. 1B and D; microparticles fabricated from each of the six formulations were spherical with smooth, non-porous surfaces. Incorporation of protein did not affect the morphology or size of the microparticles, as shown in the representative example of 85:15 PLGA with 10% w/w PLGA–PEG–PLGA (Fig. 1). The mean diameter of the microparticles formed without protein (Fig. 1A) was 90.8 μm (SD ± 24.7) and the mean diameter of microparticles containing protein (Fig. 1C) was 94.0 μm (SD ± 27.6). Multiple batches formed under the same conditions demonstrated repeatable distributions; in each case, the double emulsion process produced a bell curve size distribution.

Incorporation of HSA with lysozyme improved overall protein encapsulation within microparticles. Initial studies with lysozyme produced microparticles with low encapsulation efficiencies (average of 13%) whereas the co-addition of HSA with lysozyme increased the encapsulation to an average of 62%.

### 3.2 Protein release

**Fig. 2:**
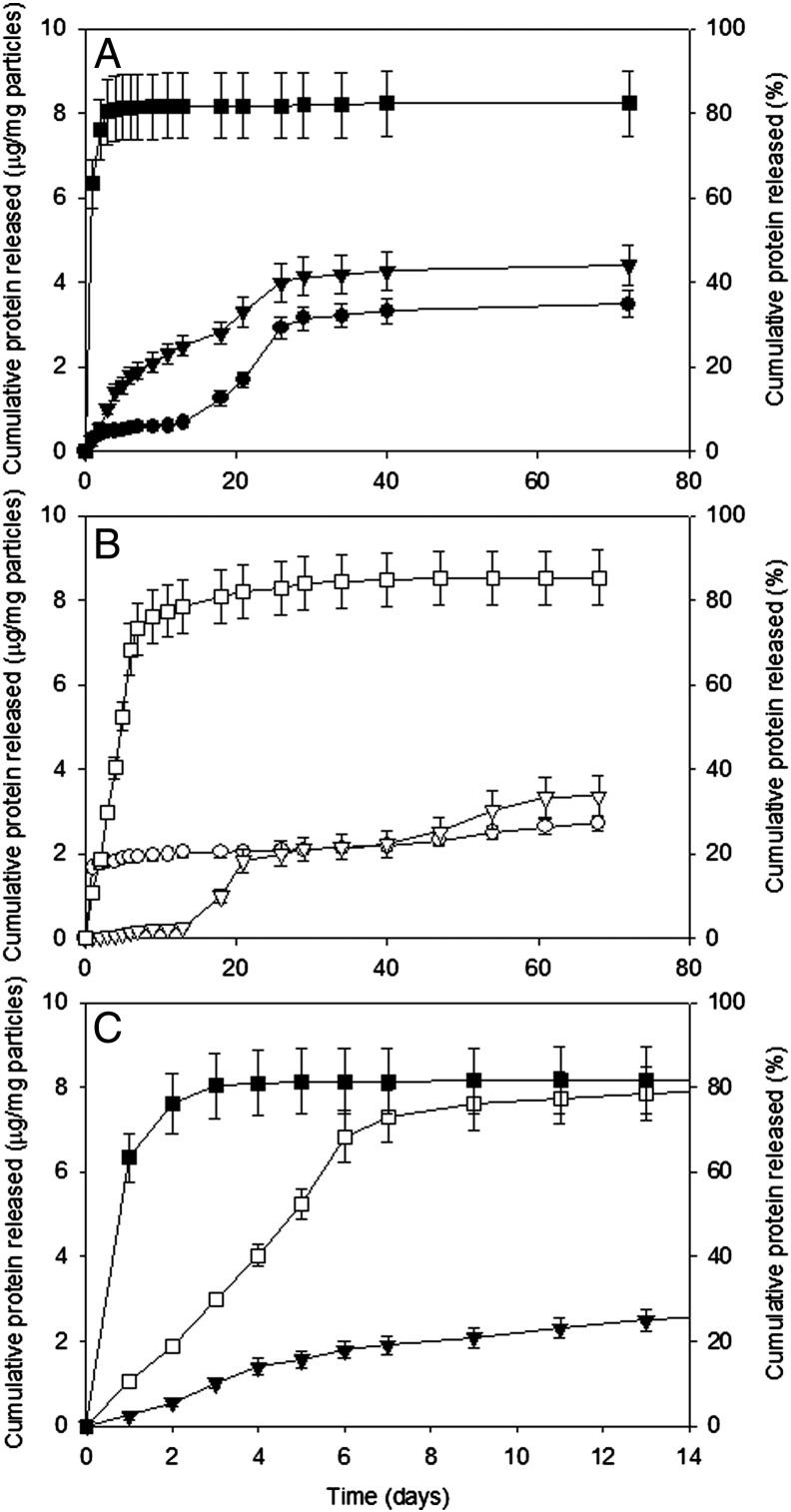
Cumulative release of HSA/lysozyme (1% w/w) from microparticles formulated from (A) 50:50 PLGA with 30% (■), 10% (▼) and 0% (●) of a PLGA–PEG–PLGA copolymer and (B) 85:15 PLGA with 30% (□), 10% (▽) and 0% (○) of a PLGA–PEG–PLGA copolymer. Release profiles of 50:50 PLGA with 30% (■), 85:15 PLGA with 30% (□) and 50:50 PLGA with 10% (▼) over 14 days are shown in (C). Data represent mean ± SD for n = 3.

Cumulative protein release from microparticles of the six formulations is shown in Fig. 2. Formulations with no triblock (0% w/w PLGA–PEG–PLGA) exhibited typical triphasic release profiles comprising: (i) an initial burst occurring during the first twenty-four hours, (ii) a lag phase and (iii) a second burst at 15 days (Fig. 2A). For 50:50 PLGA formulations the addition of PLGA–PEG–PLGA modified the release profile. A quasi zero order release profile was obtained for 50:50 PLGA 10% w/w PLGA–PEG–PLGA during the first four weeks of release. Incorporation of 30% w/w PLGA–PEG–PLGA with 50:50 PLGA increased protein release during the burst phase, with more than 60% of protein released in the first twenty-four hours. The burst phase from particles made with 85:15 PLGA was also modulated by the addition of 30% w/w PLGA–PEG–PLGA (Fig. 2B) and in both 50:50 and 85:15 formulations, more than 80% of the total protein was released (Fig. 2A and B). A triphasic release profile was exhibited by 85:15 PLGA with both 0% and 10% w/w PLGA–PEG–PLGA (Fig. 2B).

Focusing in on the first 14 days of release (Fig. 2C) shows that sustained release with a zero order profile was obtained from the 85:15 PLGA 30% w/w PLGA–PEG–PLGA formulation over this timeframe. This was in contrast to both the rapid release (approximately three days) of protein from the 50:50 PLGA 30% w/w PLGA–PEG–PLGA and the slow, steady release from 50:50 PLGA 10% w/w PLGA–PEG–PLGA.

Analysis of the average daily release of protein from the various formulations (Table 2) reveals that the 50:50 10% w/w PLGA–PEG–PLGA provides a sustained total protein release of 1.8 μg per day in the fifth week, corresponding to 180 ng/day of lysozyme. Lysozyme release from the 85:15 PLGA 30% w/w PLGA–PEG–PLGA formulation is similar (150 ng/day) whereas initial rapid protein release is evident in the higher burst values and reduced lysozyme release (40 ng/day) in the fifth week. The activity of released lysozyme was determined using the *M. lysodeikticus* assay which confirmed that the released lysozyme was active at all timepoints (data not shown).

### 3.3 Protein release from blended batches

**Supplementary Fig. 1:**
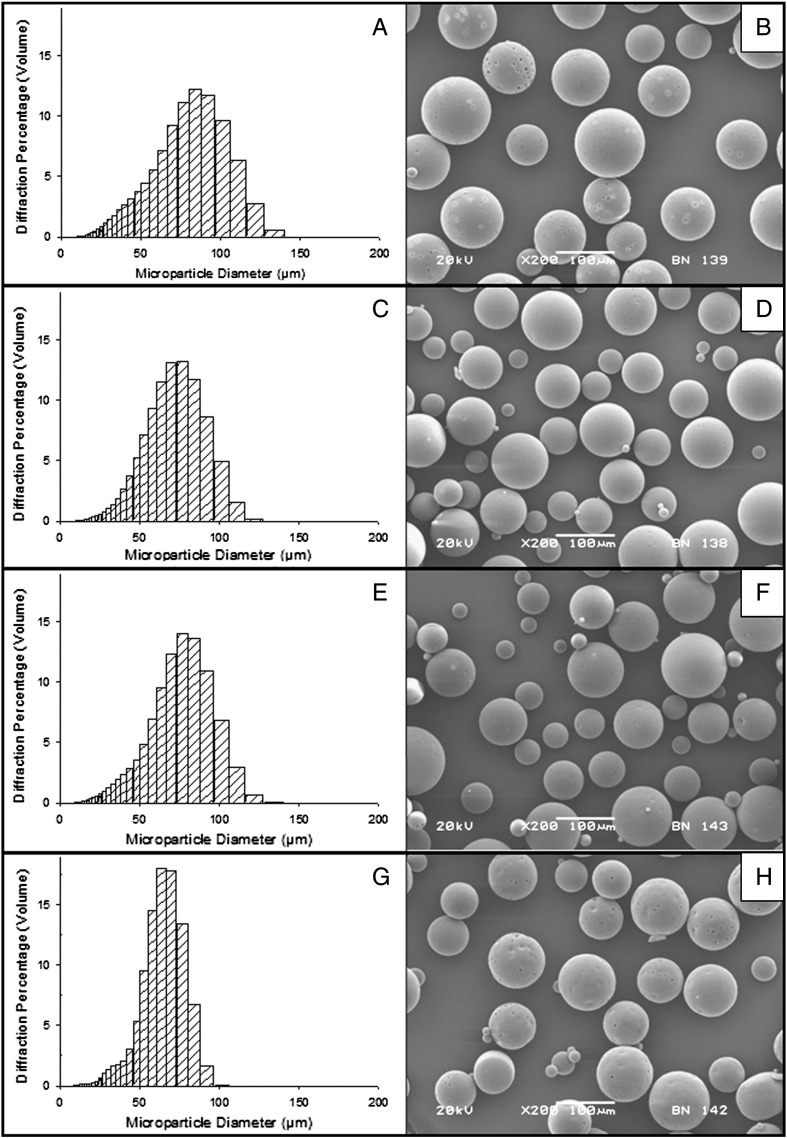
Size distributions (left) and SEM images (right) of protein loaded (1% w/w) microparticles formulated from 50:50 PLGA and a PLGA–PEG–PLGA copolymer. Concentrations of the PLGA–PEG–PLGA component were varied: 0% (A and B), 10% (C and D), 20% (E and F) and 30% (G and H).

Additional batches of microparticles were fabricated from 50:50 PLGA with 0%, 10%, 20% and 30% w/w PLGA–PEG–PLGA. Three batches of each formulation were prepared and the size, morphology and in vitro release profiles were characterised. Representative images and size data are provided in Supplementary Fig. 1. The addition of the triblock copolymer reduced the mean diameter of microparticles and produced more narrow size distributions (as can be seen in the left column of Supplementary Fig. 1). The mean diameter of 50:50 PLGA microparticles without triblock copolymer was 77.5 μm (SD ± 20.4) (Supplementary Fig. 1A) and the addition of 30% w/w PLGA–PEG–PLGA reduced the mean diameter to 64.6 μm (SD ± 15.6) (Supplementary Fig. 1G). Multiple batches formed under the same conditions demonstrated repeatable size distributions.

**Fig. 3:**
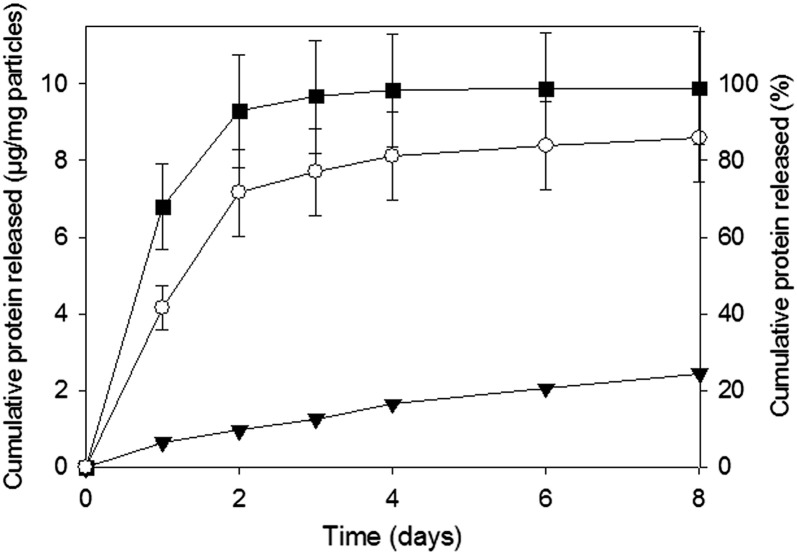
Cumulative release of HSA/lysozyme (1% w/w) from microparticles formulated from 50:50 PLGA with 30% (■) and 10% (▼) of a PLGA–PEG–PLGA copolymer and a mixture of these two formulations (1:1 ratio) (○). Data represent mean ± SD for n = 9.

Microparticles from the 50:50 PLGA 10% and 30% w/w PLGA–PEG–PLGA formulations were mixed in a 1:1 ratio to assess the effect of batch blending upon the release profile. Mixing a slow releasing formulation (containing 10% w/w PLGA–PEG–PLGA) with a more rapid formulation (containing 30% w/w PLGA–PEG–PLGA) mitigated the burst effect and provided a more overall sustained release (Fig. 3).

### 3.4 Comparative release of lysozyme and BMP-2

**Fig. 4:**
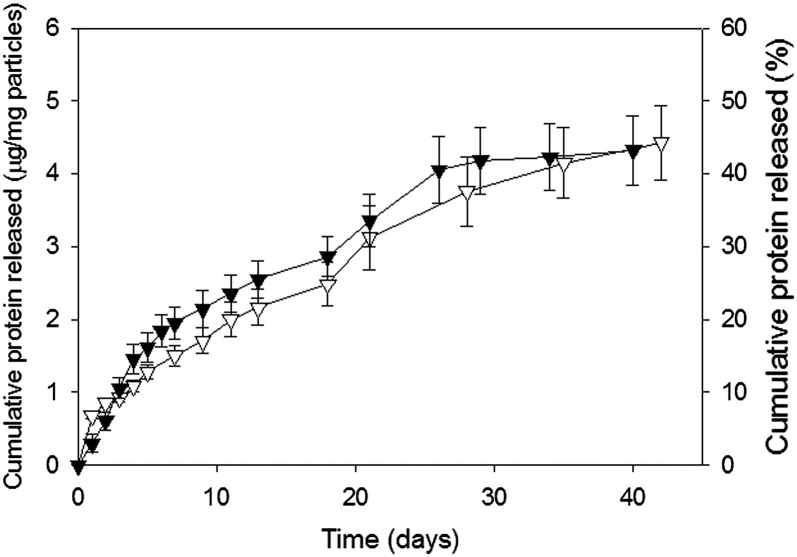
Cumulative release of HSA/lysozyme (1% w/w) (▼) and HSA/BMP-2 (1% w/w) (▽) from microparticles formulated from 50:50 PLGA with 10% w/w PLGA–PEG–PLGA copolymer. Data represent mean ± SD for n = 3.

Microparticles containing HSA/BMP-2 were fabricated from 50:50 PLGA with 10% w/w PLGA–PEG–PLGA. The release profiles of HSA/BMP-2 and HSA/lysozyme from this formulation were in accord (Fig. 4) indicating that lysozyme was a suitable model for BMP-2 in this work.

## 4 Discussion

Successful scaffolds for growth factor delivery must provide both structural support and the controlled spatio-temporal release of growth factors required to host tissue formation. Although spatio-temporal control of growth factor and protein delivery has been achieved previously via encapsulation within polymeric microparticles, providing appropriate release profiles for the protein or growth factor of interest remains a challenge [16]. Protein release profiles consisting of an initial burst and subsequent slow and incomplete release (that does not match polymer degradation rate) have often been observed and are deemed inappropriate for clinical use [31–35]. In addition, incomplete release has been linked to protein instability issues that may occur during formulation, storage and release of protein from loaded microspheres [31,36,37].

Thus, the objective of this study was to develop a novel delivery system, capable of providing structural support, with controlled and tuneable release kinetics. Our strategy was to include a hydrophilic polymer within microparticle formulations. Protein delivery systems based on hydrophilic–hydrophobic block copolymers have been utilised as controlled release systems since the degradation rate, and hence release kinetics, can be modulated by adjusting copolymer compositions [24,25,38–40]. In particular, copolymers of PLGA and PEG are more compatible with proteins, reduce protein adsorption and favour homogeneous protein distribution within the polymer matrix as well as increasing water uptake in microspheres [41].

In this study we show, for the first time, that protein release kinetics can be altered and controlled by the inclusion of the PLGA–PEG–PLGA triblock copolymer and that these release kinetics can be decoupled from polymer degradation rate. Subsequently, we have developed a novel delivery system with capability for delivering multiple proteins with different and independent release kinetics.

The addition of the PLGA–PEG–PLGA copolymer modulated protein release by accelerating water ingress into the matrix. Increased water uptake can lead to a combined mechanism of swelling and structural erosion, which may produce a hydrogel-like structure in the microparticles, facilitating controlled release of the protein. Addition of 10% w/w PLGA–PEG–PLGA to 50:50 PLGA modulated the typical triphasic profile and instead produced a quasi zero order release profile over a four week duration. For both 85:15 and 50:50 PLGA, almost complete protein release was achieved with 30% w/w triblock copolymer included in the formulation; sustained release over ten days was achieved with 85:15 PLGA 30% w/w PLGA–PEG–PLGA. These three formulations provide diverse and independent release profiles that could be used for the delivery of growth factors. For example, the rapid release from 50:50 PLGA 30% w/w PLGA–PEG–PLGA may be suitable for delivering VEGF (to stimulate immediate blood vessel formation) whereas the more sustained release of the 85:15 PLGA 30% w/w PLGA–PEG–PLGA formulation may be appropriate for PDGF (to promote maturation of the newly formed blood vessels). The sustained duration of release of HSA/lysozyme from 50:50 PLGA 10% w/w PLGA–PEG–PLGA renders this an ideal delivery vehicle as BMP-2 is typically expressed throughout the fracture healing process until remodelling occurs (21 days) [42]. The kinetics of release of rhBMP-2 have recently been shown to have profound effects on bone regeneration; an initial burst followed by sustained release significantly improved healing and bone regeneration in rat femurs [43,44]. Furthermore, the average daily release values of lysozyme, reported in Table 2, correspond to values reported for BMP-2 delivery for effective bone repair [45–47].

For microparticles fabricated with 50:50 PLGA, the onset of polymer degradation occurred at approximately day 15. This resulted in a second burst of protein release in the triphasic release profile of the 50:50 PLGA 0% w/w PLGA–PEG–PLGA microparticles. With 50:50 PLGA 30% w/w PLGA–PEG–PLGA, we were able to achieve almost complete release in five days, i.e. release occurred prior to the onset of polymer degradation. Analogous behaviour was observed with 85:15 PLGA 30% w/w PLGA–PEG–PLGA with kinetics of release decoupled from polymer degradation rate. Although incomplete release was observed for the other four formulations, total protein release was higher in the 10% w/w PLGA–PEG–PLGA formulations compared to those fabricated from PLGA alone. This suggests that the presence of the triblock copolymer may provide a means to address this phenomenon.

The kinetics of release were also modified by mixing microparticles of two different formulations providing another route, not previously reported, for controlling release kinetics. Mixing a slow releasing formulation (containing 10% w/w PLGA–PEG–PLGA) with a more rapid formulation (containing 30% w/w PLGA–PEG–PLGA) mitigated the burst effect and provided a sustained release profile.

In this study, protein entrapment occurred via a double emulsion process. The creation of a water/organic solvent interface during the primary emulsion stage has been well documented in the literature as being primarily responsible for protein instability during emulsification [36,48,49]. However, in our study, good encapsulation efficiencies and retention of protein activity was observed. This is likely to be due to our approach of adding an excipient, (HSA in our case) to the inner aqueous phase that can compete with the therapeutic protein at the water/organic solvent interface. We chose HSA for our work based on publications where serum albumins in particular have been shown to limit emulsification induced aggregation of several different proteins, including lysozyme, ovalbumin and recombinant human erythropoietin [49–51]. HSA was co-encapsulated with lysozyme (in a ratio of 9:1 HSA:lysozyme) which did indeed appear to shield lysozyme from interface induced aggregation evidenced by retention of enzymatic activity but also increased the encapsulation efficiency. A similar effect was observed by Srinivasan et al. [51] with rat serum albumin.

Lysozyme was used as a model protein in this work due to the breadth of detailed characterisation of this protein present in published literature [48,49,52–56] coupled with the ability to easily measure biological activity [57]. An additional motivation for using lysozyme was that it is considered an appropriate model for BMP-2 due to the similarity of isoelectric points (lysozyme: 9, BMP-2: > 8.5) and molecular weights (lysozyme: 14 kD, BMP-2: 26 kD) [26]. The consistency shown in the release profiles of HSA/BMP-2 and HSA/lysozyme confirms this. Previous work from our group with microspheres formulated as described above verified the biological activity of released BMP-2 from PLGA microparticles formulated with PLGA–PEG–PLGA [23].

## 5 Conclusions

A new delivery system, capable of providing controlled release kinetics, has been developed from PLGA microparticles. The inclusion of a hydrophilic PLGA–PEG–PLGA triblock copolymer altered release kinetics such that they were decoupled from polymer degradation. A quasi zero order release profile over four weeks was produced using 10% w/w PLGA–PEG–PLGA with 50:50 PLGA whereas complete and sustained release over was achieved over ten days using 30% w/w PLGA–PEG–PLGA with 85:15 PLGA and over four days using 30% w/w PLGA–PEG–PLGA with 50:50 PLGA. These three formulations are promising candidates for delivery of growth factors such as BMP-2, PDGF and VEGF. Release profiles were also modified by mixing microparticles of two different formulations providing another route, not previously reported, for controlling release kinetics. Good encapsulation efficiencies and retention of protein activity were achieved via the co-addition of HSA with lysozyme. Comparative release of HSA/lysozyme and HSA/BMP-2 showed excellent agreement confirming that lysozyme is an appropriate model for BMP-2.

The following are the supplementary data related to this article.

## Figures and Tables

**Table 1:** Triblock copolymer characteristics.

*M_N_*^a^	% mole lactide^a^	% mole glycolide*^a^*	*M_N_*^b^	*M_W_*^b^	*PDI*^b^
1706–1500–1706	71	29	2442	4022	1.65

^a^determined by ^1^H NMR; ^b^determined by GPC.

**Table 2:** Average daily release of HSA/lysozyme (1% w/w) from three microparticle formulations; microparticle mass = 100 mgs.

Days	Average daily release HSA/lysozyme (μg)		
	50:50 PLGA 10% PLGA–PEG–PLGA	85:15 PLGA 30% PLGA–PEG–PLGA	50:50 PLGA 30% PLGA–PEG–PLGA
1–7	27.1	104.5	116.3
8–14	9.4	8.2	0.3
15–21	10.7	4.6	0.0
22–28	10.9	2.0	0.2
29–35	1.8	1.5	0.4
